# LGBTQIA+ AD/ADRD CAREGIVERS’ PERCEPTIONS OF THE ACCEPTABILITY OF A LEGAL AND FINANCIAL PLANNING TRAINING TOOL

**DOI:** 10.1093/geroni/igae098.4230

**Published:** 2024-12-31

**Authors:** Clover Caldwell, Richard Chunga, Sydney Hoel, Heather Schwartz, A J Slowey, Nicole Werner, Andrew Pickett

**Affiliations:** Indiana University Bloomington, Bloomington, Indiana, United States; University of Massachusetts Boston, Boston, Massachusetts, United States; Indiana University, Bloomington, Bloomington, Indiana, United States; Indiana University Bloomington, Bloomington, Indiana, United States; Indiana University Bloomington, Bloomington, Indiana, United States; Indiana University, Bloomington, Bloomington, Indiana, United States; Indiana University, Bloomington, Bloomington, Indiana, United States

## Abstract

LGBTQIA+ identifying informal caregivers for people living with Alzheimer’s Disease (AD) and AD-related dementias (AD/ADRD) face multiple, overlapping stressors related to identity stigmatization and role-based burden. This includes legal/financial planning, as the legal landscape surrounding LGBTQIA+ identity is inconsistent and ever-changing. Part of a larger project, this study examined LGBTQIA+ identifying caregivers’ perceptions of the acceptability of CareVirtue Planner, a web-based legal and financial planning support tool for AD/ADRD caregivers. Semi-structured interviews with LGBTQIA+ caregivers (n=9) were conducted after three months of use. We conducted an inductive content analysis using the following process: open coding, collaborative generation of the initial codebook, dual coding with the codebook, consensus discussion on refinements to finalize codebook. Our analysis resulted in four overarching themes among users: sense of commonality and personal validation related to legal and financial challenges of caregiving, Planner features promoting caregiver preparedness, inclusive language and need for LGBTQIA+ specific tailoring, and individual characteristics and personal histories impacting relative usefulness of the Planner. Participants broadly found Planner material to be well organized and its features useful (e.g., document storage ‘vault’), with content best suited for those early in the caregiving journey. Users provided specific recommendations to increase LGBTQIA+ appropriateness, including creation of additional content to specifically address unique legal challenges facing LGBTQIA+ caregivers, inclusion of diverse families in materials (e.g., vignettes), and vetting of externally linked resources for LGBTQIA+ inclusiveness. Further research is needed to support LGBTQIA+ AD/ADRD caregivers navigating complex legal and financial situations.

